# Multiport anterior retroperitoneal access for robotic-assisted partial nephrectomy: an innovative technique for renal tumour

**DOI:** 10.3389/fsurg.2025.1630319

**Published:** 2025-10-15

**Authors:** Tommaso Silvestri, Sara Riolo, Bernardino De Concilio, Guglielmo Zeccolini, Giovanni Costa, Damiano D'Aietti, Roberto Knez, Antonio Celia

**Affiliations:** 1 Department of Urology, San Bassiano Hospital, Bassano del Grappa, Italy; 2 Department of Urology, Sapienza University of Rome, Rome, Italy

**Keywords:** partial nephrectomy, robotic partial nephrectomy, retroperitoneal, retroperitoneal partial nephrectomy, retroperitoneal access, retroperitoneal region, kidney cancer, localized kidney cancer

## Abstract

Robotic Partial Nephrectomy can be performed via transperitoneal or retroperitoneal access. The retroperitoneal approach offers direct access to the renal artery and reduces the risk of intestinal injury, especially in patients with prior abdominal surgeries or adhesions. This study presents a novel retroperitoneal robotic technique using anterior trocar placement with the patient in lateral decubitus. Access is gained through a pararectal incision, with the AirSeal trocar placed first, followed by the creation of an extraperitoneal working space and placement of robotic trocars. After docking, the renal hilum is isolated, the mass is enucleated following arterial clamping, and the collecting system is sutured selectively. The procedure ends with hemostasis and drainage tube placement. This is the first reported use of anterior retroperitoneal access with the patient in lateral decubitus, differing from previous techniques performed in the supine position. Advantages include avoiding intraperitoneal adhesions and improved assistant access. The technique is especially suitable for renal tumors in the middle or lower third of the kidney but can also be applied to upper pole lesions. Preliminary outcomes are promising; further research with larger cohorts is needed to validate its effectiveness.

## Introduction

Renal Cell Carcinoma represents the 14th-most frequently diagnosed cancer and approximately 60% of renal cell carcinomas are detected incidentally on abdominal imaging (1).

Current European Guidelines recommend Partial Nephrectomy (PN) for patients with T1 tumors and for patients with T2 tumors and a solitary kidney or chronic disease, if technically feasible. Technological improvements have paved the way for the gradually increased employment of minimally invasive approaches, first with the advent of laparoscopy and then with robotics, progressively minimizing the impact and invasiveness of the surgical procedure.

Robotic Partial Nephrectomy is possible with both transperitoneal (TP) or retroperitoneal (RP) access and recently, several studies have compared these two techniques (2, 3) and many classification systems and scores have been designed to guide the surgeon's choice between transperitoneal and retroperitoneal access (4). Transperitoneal access is more frequent and urologists are more familiar with the anatomical references and spatial orientation of the transperitoneal route but the retroperitoneal offers direct access to the renal artery, it preserves the intraperitoneal space, and carries a lower theoretical risk of intestinal lesions, especially in patients with multiple previous surgeries and when there is suspicion of multiple intestinal adhesions, and the intact peritoneum prevents urine spillage into the peritoneal cavity if urinoma formation occurs postoperatively (5). Historically, the RP approach has been utilized principally to address posterolateral lesions as it has a fairly direct line of sight to these surfaces of the kidney. However, the scope of the RP approach has gradually expanded to include the extirpation of masses in other regions of the kidney (6) and studies have found that the location of tumor (anteriorly or posteriorly) does not influence perioperative outcomes following retroperitoneal robotic-assisted partial nephrectomy (RP-RAPN) (7).

Nevertheless, the RP approach is more challenging due to the confined space and less familiar landmarks. Furthermore the TP approach has a less steep learning curve as compared to the RP approach, where developing the RP space and working in it can be challenging and may lead to an increased risk of renal vascular injuries (8). On the contrary, some studies suggest that the learning curve for RP access is not long or complicated, and that, in surgeons who are still learning, surgical outcomes for RP access are immediately comparable to those of TP access (9).

Several variations of the surgical technique for RP access have been described based on the location of the tumor and the characteristics of the patient (10). The aim of this study is to present a new surgical technique for RP-RAPN. The M.A.R.A. (Multiport Anterior Retroperitoneal Access) technique involves an anterior trocar arrangement for accessing renal masses located in the middle or lower third of the kidney, although masses located near the upper pole can also be easily treated. The main advantage is that it allows the assistant to work more easily compared to the standard RP setup, facilitating RP access to various types of renal masses, including those located anteriorly, with the aim of preserving the integrity of the peritoneal cavity. This technique represents a significant advancement in the approach to robotic retroperitoneal partial nephrectomies, offering benefits in terms of both visibility and convenience for the surgical team.

A similar access has already been described for single-port surgery by Pellegrino et al. (11), whose technique is summarized by the acronym SARA (Supine Anterior Retroperitoneal Access). This refers to a single-port surgery developed to overcome the bulky robotic structure in the limited retroperitoneal space and the potential resulting instrument clashing. Although the type of access is similar, our MARA technique differs from SARA by employing a multiport robotic surgery approach. It proposes a trocar arrangement designed to overcome the aforementioned logistical challenges of operating in a confined space and to facilitate access to masses that would otherwise be difficult to manage via the retroperitoneal approach.

The clinical outcomes of patients treated with this technique so far at our center have proven to be comparable to those reported in the literature for RP-RAPN (12). Surgical times, intraoperative blood loss, positive surgical margins, complications according to the Clavien-Dindo classification, and length of hospital stay for the first 18 patients treated with this technique are summarized in Table 1. However, comprehensive statistical analyses and further studies will be performed on a larger patient cohort, obviously taking into account patient characteristics and tumor features by calculating the RENAL and PADUA scores, to more clearly define the role of this surgical technique in the treatment of renal lesions.

**Table 1:** The primary outcomes evaluated in the initial group of treated patients.

Outcome	Mean value
Surgical time (minutes)	135 ± 20
Intraoperative blood loss (ml)	65 ± 25
Positive surgical margins (PSM)	1/18 (5.6%)
Clavien-Dindo complications ≥3	1/18 (5.6%)
Length of hospital stay (days)	2.1 ± 0.5

## MARA technique

**Figure 1:**
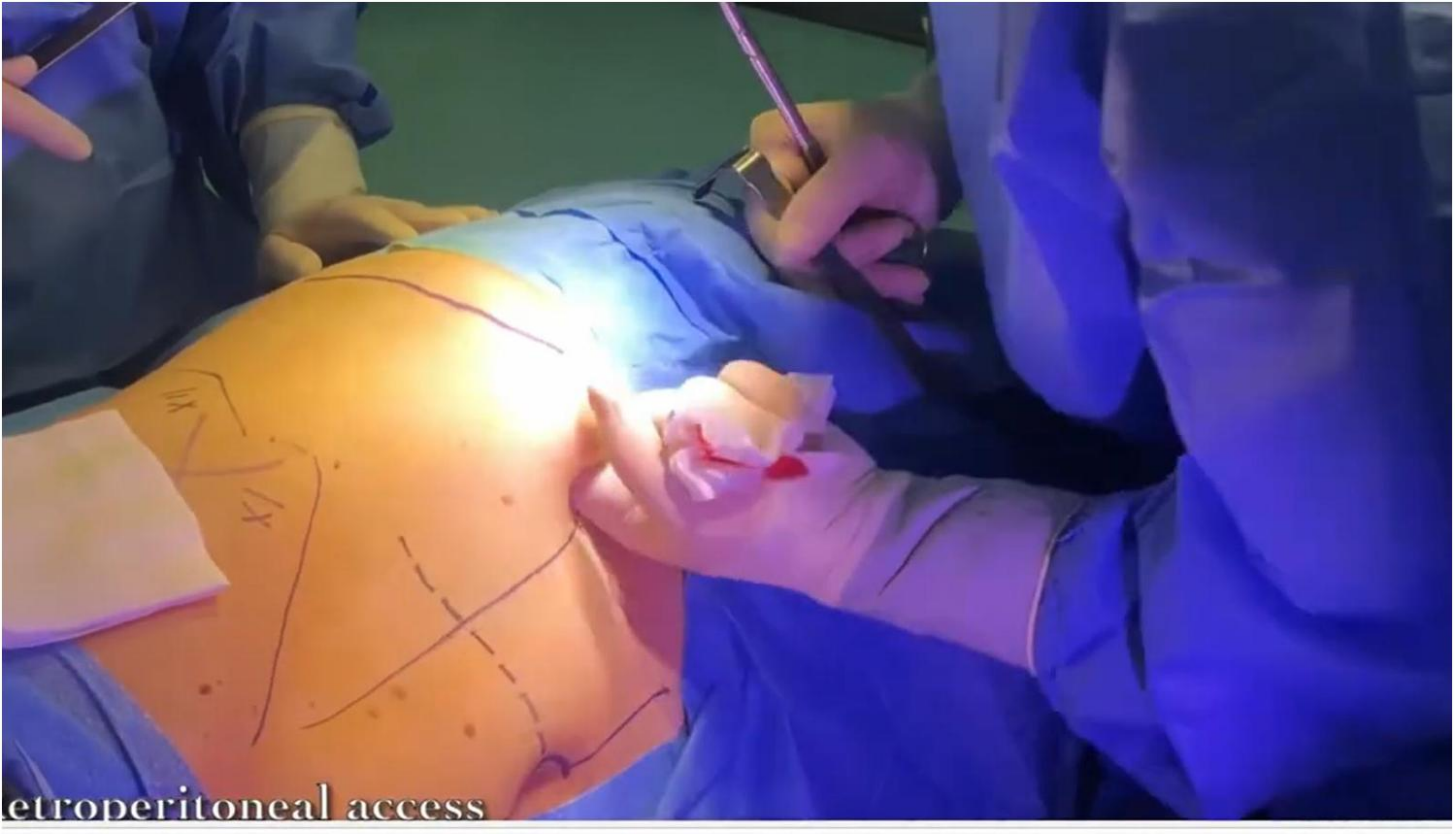
Preoperative drawing with the first access highlighted.

**Figure 2:**
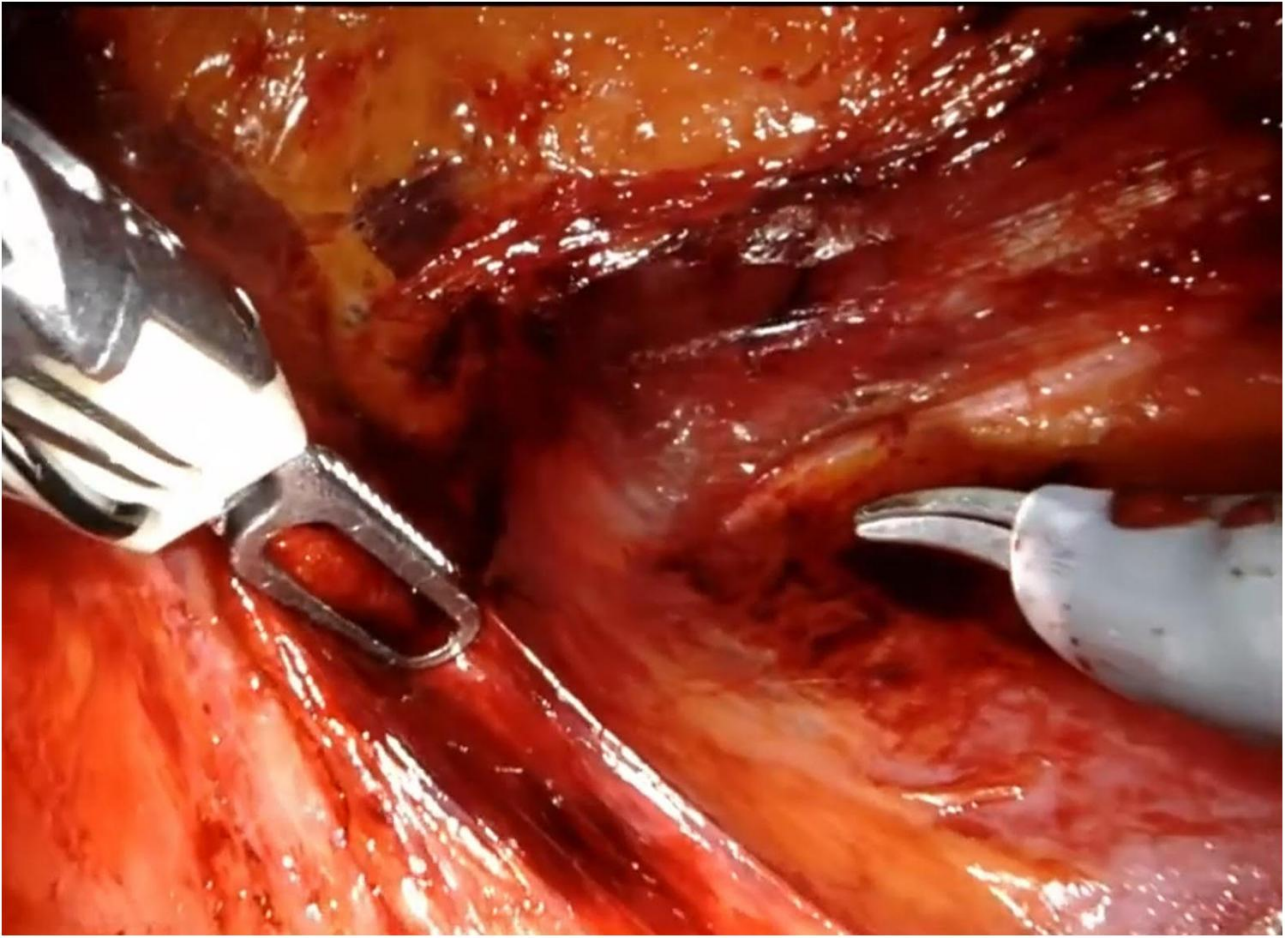
Creation of retroperitoneal space.

**Figure 3:**
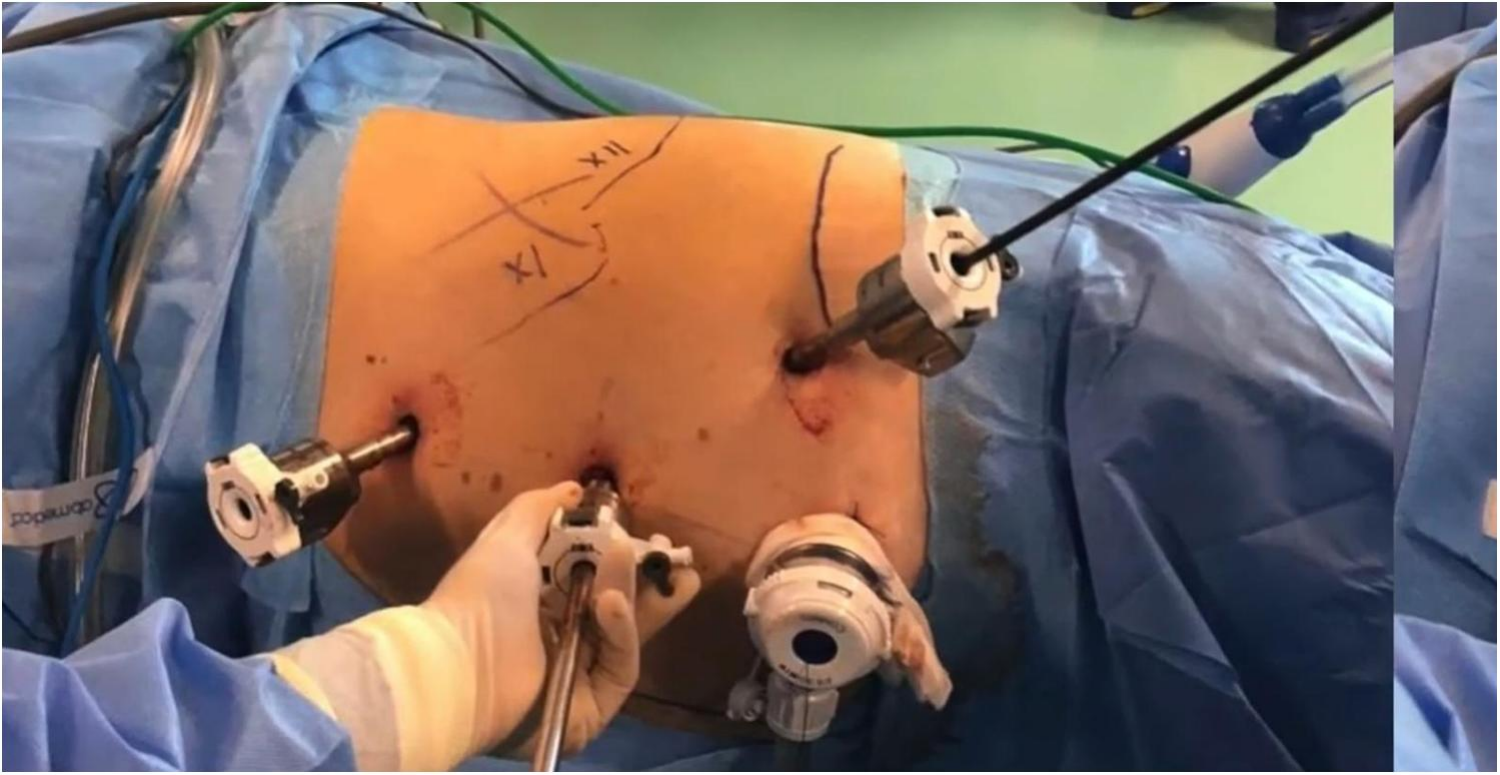
Trocar placement completed.

The patient is placed under general anesthesia and positioned on the side opposite to the lesion. An initial incision is made superior to the pararectal line on the side of the lesion, 2 cm from the anterior superior iliac spine, following the Hasson technique (Figure 1). Once the extraperitoneal space is reached, a cavity is created using a blunt approach (Figure 2), and a balloon dissector is inserted to enlarge the space. The balloon is then removed, and the trocar for the AirSeal system is placed, inducing a pneumoretroperitoneum at a pressure of 10 mmHg. The robotic endoscope is introduced through the inserted trocar, allowing identification of the psoas muscle and the peritoneal reflection. An 8 mm robotic port (for the monopolar curved scissors) is placed laterally, approximately 4 cm from the previously positioned trocar. Through this port, a Johann laparoscopic clamp is introduced to medialize the peritoneal reflection. This step is critical for creating space to place the additional robotic ports while minimizing the risk of accidental peritoneal breach. Once sufficient space is created, another 8-mm robotic port (for the fenestrated bipolar forceps) is positioned laterally, also 4 cm from the first trocar, forming a triangular configuration. Additional trocars (including a 5 mm laparoscopic port) are placed along a line approximately 6 cm from the previously inserted ports to optimize access and maneuverability during the procedure (Figure 3).

For masses located anteriorly or involving the upper pole, the AirSeal trocar is used for the table assistant's laparoscopic instruments. Otherwise, docking is performed with this configuration (optics inside the AirSeal trocar).

**Figure 4:**
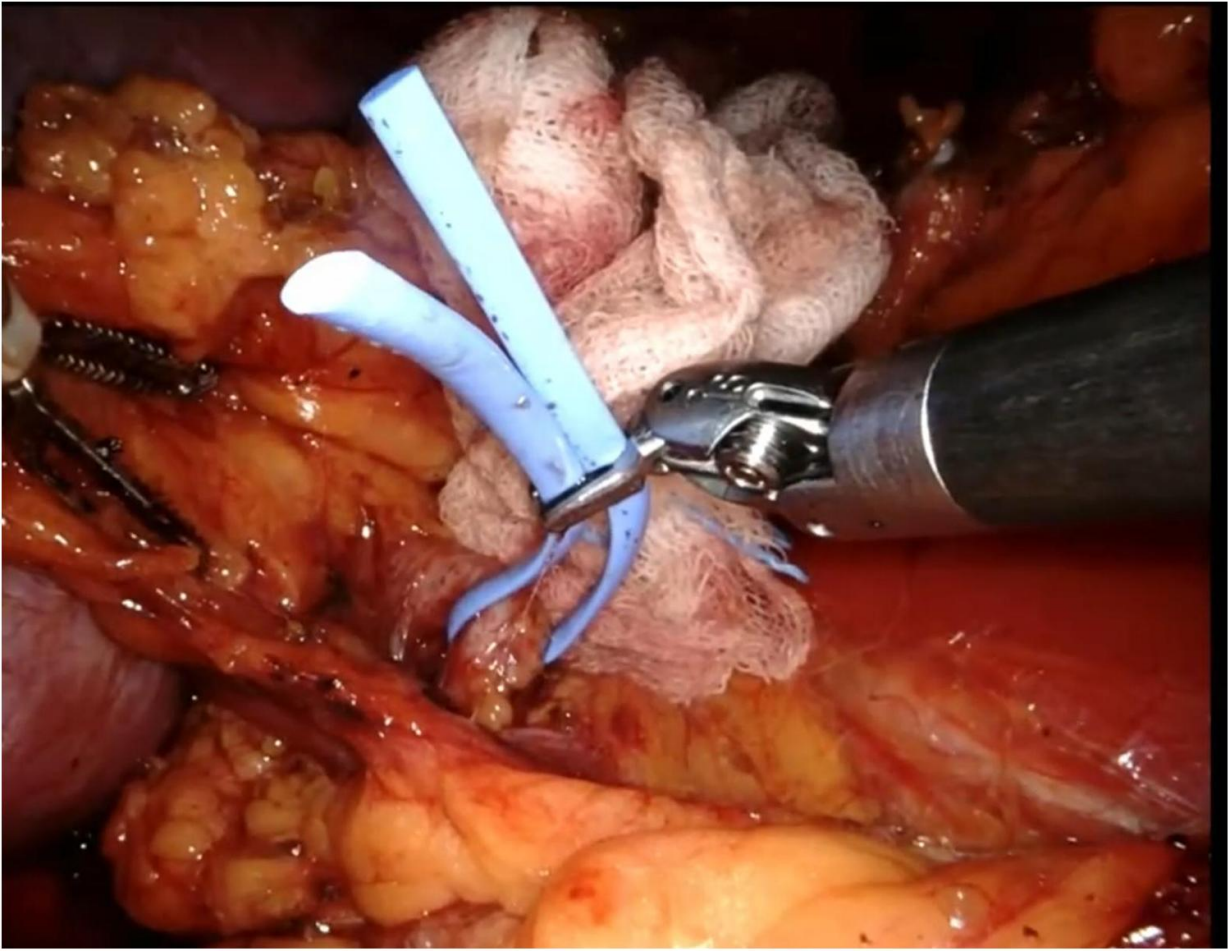
The renal artery is identified and marked by placing a vessel loop.

**Figure 5:**
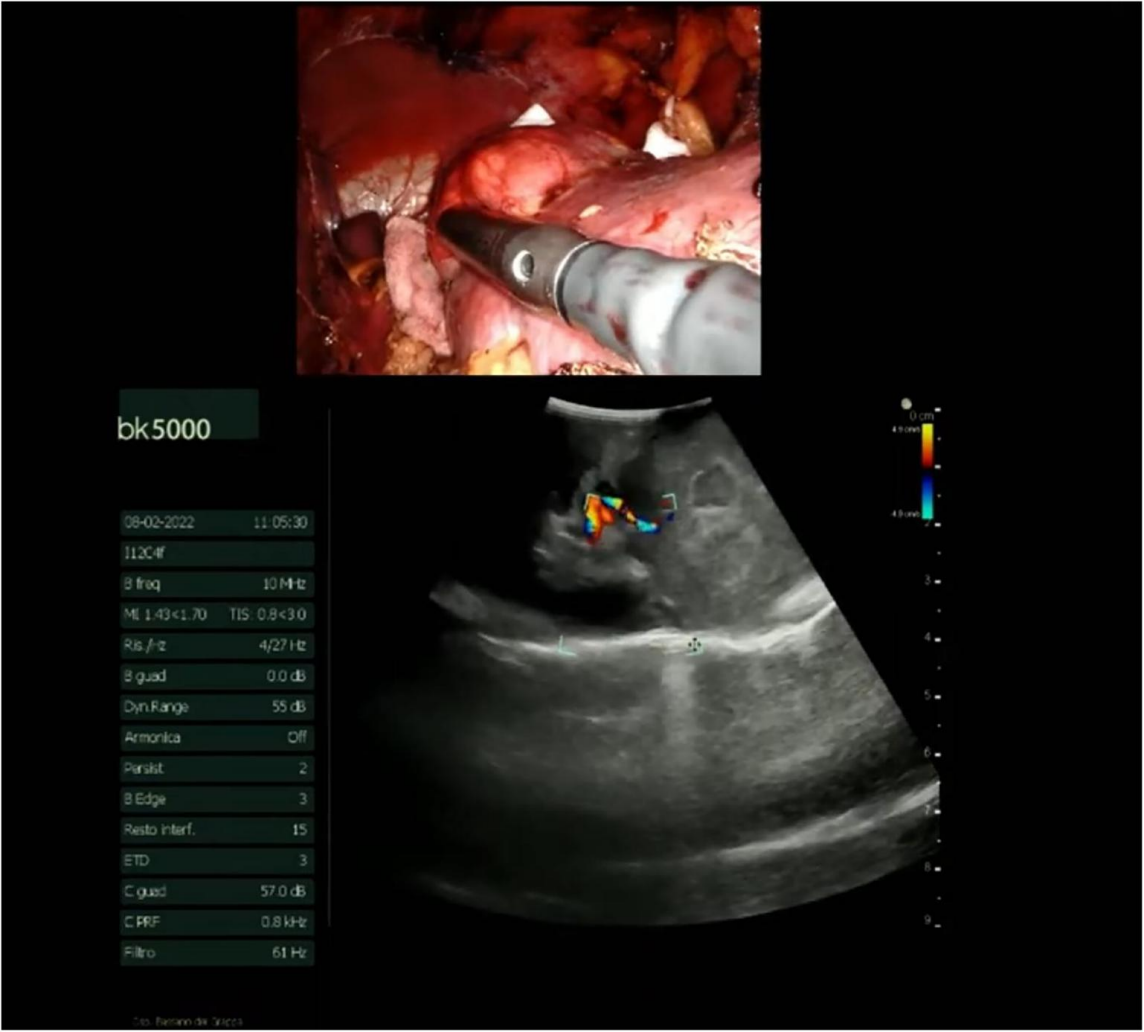
Intraoperative ultrasound.

**Figure 6:**
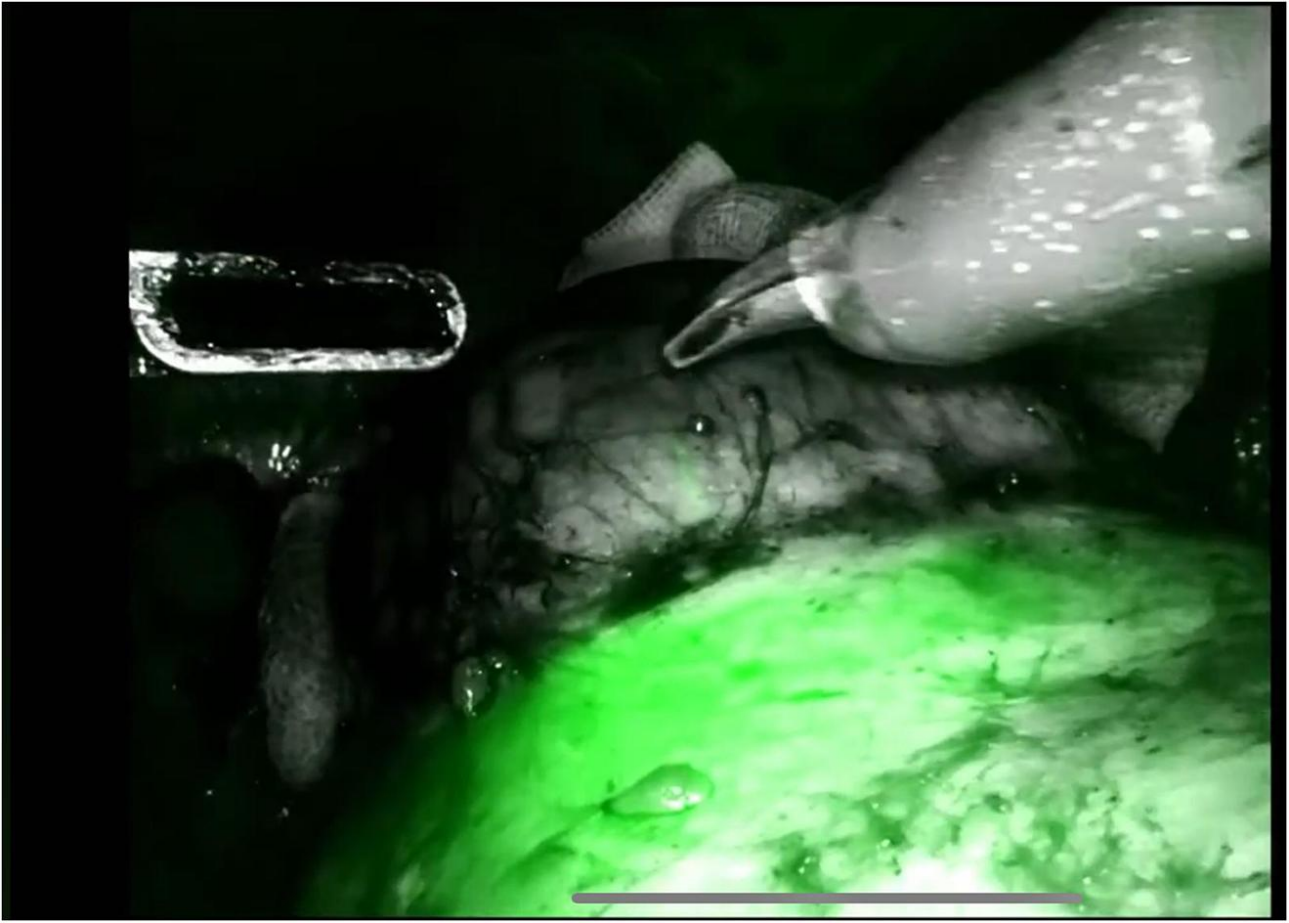
Use of indocyanine green (ICG).

The procedure begins with an incision of Gerota's fascia, followed by identification of the mass to be removed. The ureter is located, and by following it, the renal hilum is reached. The renal artery is isolated, and a vessel loop is placed in position (Figure 4). Depending on the lesion's characteristics (size, vascularization) and the patient's condition, the renal artery may be clamped or left unclamped. Both approaches are feasible and safe. Intraoperative ultrasound is used to assess the lesion's location, depth within the renal parenchyma, and other characteristics (Figure 5). Indocyanine green is administered intravenously to monitor ischemia (in clamped cases) and vascularization (Figure 6).

**Figure 7:**
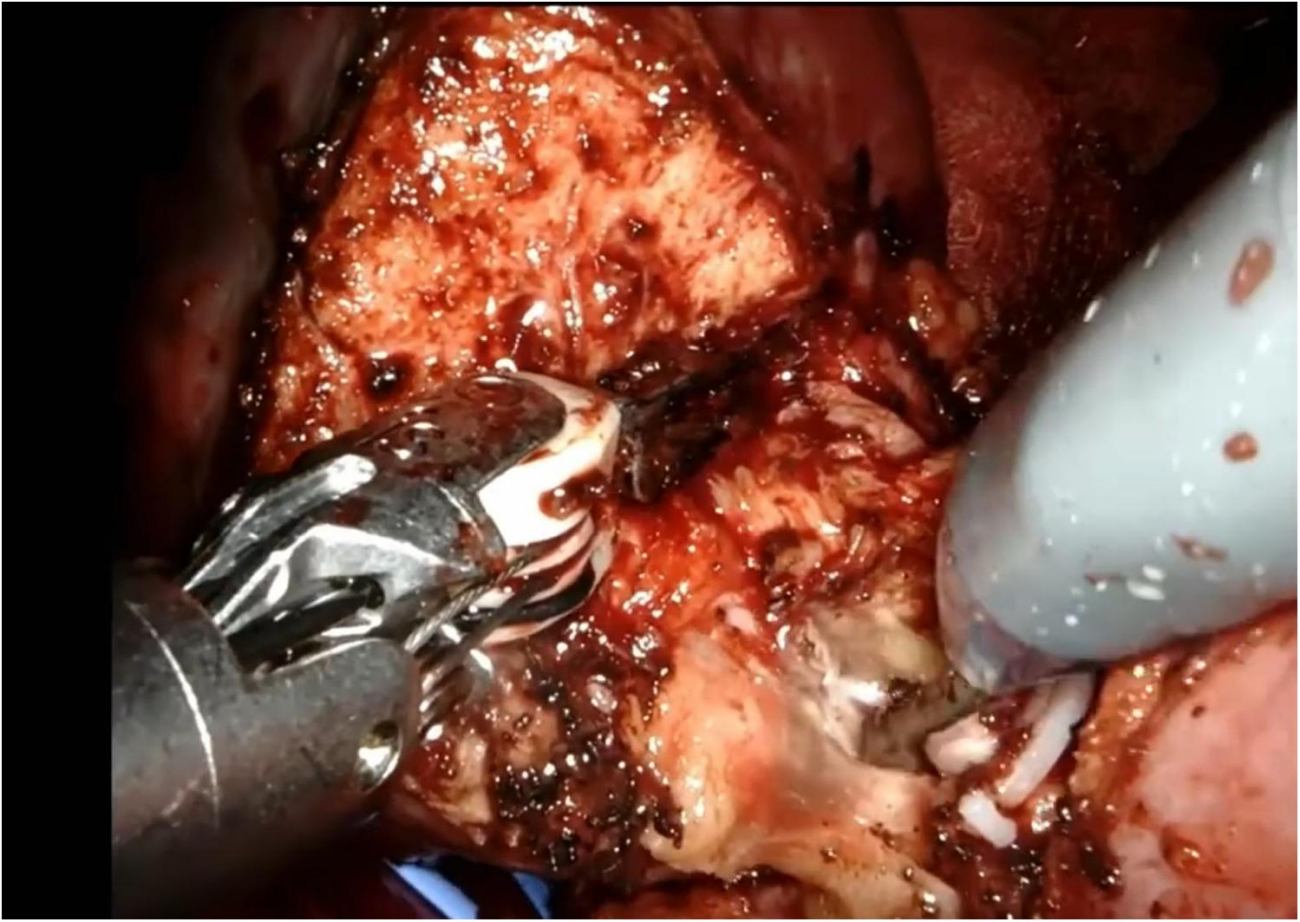
Enucleation phase.

**Figure 8:**
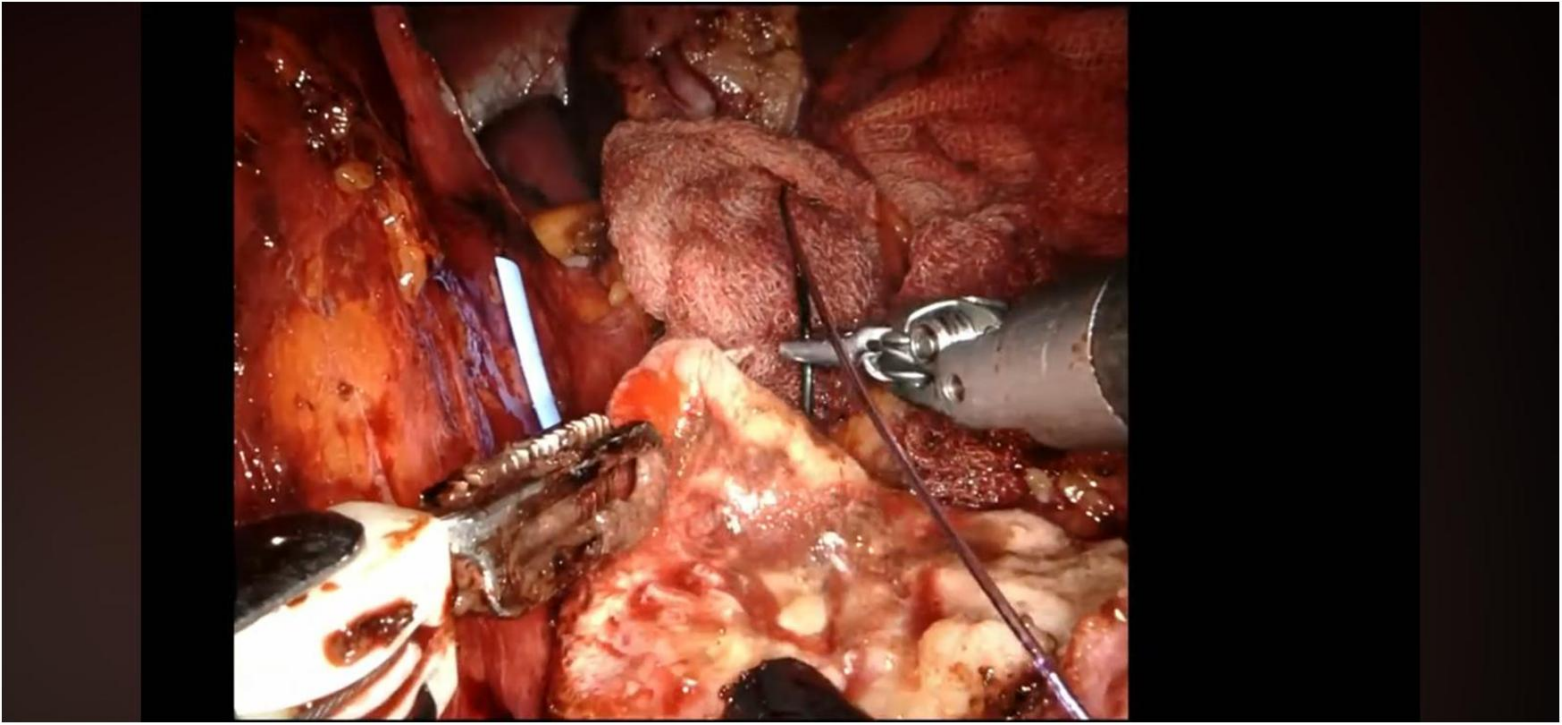
Renorrhaphy of the renal parenchyma.

The renal mass is progressively enucleated using the robotic monopolar scissors (Figure 7). The lesion is placed inside an Endobag. Once enucleation is complete, hemostasis is controlled at the resection bed. A sliding clip renorrhaphy is performed to close the breach in the renal parenchyma, with suturing carried out in multiple stages (Figure 8). Any openings of the urinary tract are sutured separately or together with the medullary tissue using a Monosyn 3/0 suture. For cortical tissue, suturing is performed with a Vicryl 2/0 suture.

In cases of large renal masses with significant tissue loss, the Lapra-Ty system may be useful. In contrast, for small exophytic masses, a sutureless approach can be used, with the resection bed coagulated by monopolar scissors, followed by the application of Tachosil or similar devices (13).

If the clamp technique was used, the renal artery is declamped, and careful hemostasis is checked. The vessel loop is then removed from the renal artery. The specimen is then removed through the AirSeal port, and a drainage tube is placed in selected cases. Finally, the robotic ports are closed.

## Conclusion

Our goal is to present a modified RP approach for the surgical treatment of renal masses located particularly in the middle third of the kidney or at the lower pole, however, it is extendable to renal masses located anteriorly and renal masses located at the upper pole. The anterior positioning of the trocars simplifies the procedure and facilitates the assistant's role, improving access to the mass for removal. Larger case series and randomized studies are needed to validate this technique. Further studies will assess the safety of this technique and its perioperative outcomes. While the choice of RP access still depends on the preferences and habits of the surgeon, the type of RP access and the placement of the trocars should be based on the anatomical characteristics of the lesion, and the ease with which it can be approached using the robotic and laparoscopic arms of the assistant should be evaluated (14).

## Data Availability

The original contributions presented in the study are included in the article/Supplementary Material, further inquiries can be directed to the corresponding author.
